# Live-Imaging Readouts and Cell Models for Phenotypic Profiling of Mitochondrial Function

**DOI:** 10.3389/fgene.2019.00131

**Published:** 2019-03-01

**Authors:** Eligio F. Iannetti, Alessandro Prigione, Jan A. M. Smeitink, Werner J. H. Koopman, Julien Beyrath, Herma Renkema

**Affiliations:** ^1^Khondrion BV, Nijmegen, Netherlands; ^2^Department of Biochemistry, Radboud Institute for Molecular Life Sciences, Radboud University Medical Center, Nijmegen, Netherlands; ^3^Max Delbrück Center for Molecular Medicine, Berlin, Germany; ^4^Radboud Center for Mitochondrial Medicine, Radboud University Medical Center, Nijmegen, Netherlands

**Keywords:** mitochondrial disease, pathological phenotype, cell models of disease, assay development, live cell microscopy, fluorescent probes, HCS, cellomics

## Abstract

Mitochondria are best known as the powerhouses of the cells but their cellular role goes far beyond energy production; among others, they have a pivotal function in cellular calcium and redox homeostasis. Mitochondrial dysfunction is often associated with severe and relatively rare disorders with an unmet therapeutic need. Given their central integrating role in multiple cellular pathways, mitochondrial dysfunction is also relevant in the pathogenesis of various other, more common, human pathologies. Here we discuss how live-cell high content microscopy can be used for image-based phenotypic profiling to assess mitochondrial (dys) function. From this perspective, we discuss a selection of live-cell fluorescent reporters and imaging strategies and discuss the pros/cons of human cell models in mitochondrial research. We also present an overview of live-cell high content microscopy applications used to detect disease-associated cellular phenotypes and perform cell-based drug screening.

## Introduction

Mitochondrial diseases can result from mutations in the nuclear (nDNA) or mitochondrial DNA (mtDNA). These mutations often lead to disruption of mitochondrial function and/or (ultra) structure leading to “primary mitochondrial disorders” (Koopman et al., 2012) that are progressive, multisystemic and relatively rare (prevalence ∼1:5000) (Parikh et al., 2015). Mitochondrial dysfunction is also a hallmark of various neurodegenerative disorders like Alzheimer’s, Parkinson’s, and Huntington’s disease, cardiovascular disease, cancer, diabetes, and epilepsy (Koopman et al., 2012). Mitochondrial dysfunction can also be triggered by environmental factors (Meyer et al., 2013) and off-target drug effects (Schirris et al., 2015).

Mitochondrial function operates at the junction of numerous cellular signaling and metabolic pathways. At the metabolic level, the tricarboxylic acid cycle together with the four complexes (CI-CIV) of the electron transport chain (ETC) and the F_o_F_1_-ATP synthase (or CV) converts food-derived metabolites into ATP. This process consumes oxygen (O_2_) and establishes a membrane potential (Δψ) across the mitochondrial inner membrane (Mitchell, 1961). But mitochondria are also key players in cellular redox homeostasis, calcium signaling, branched-chain amino acid metabolism and the coordination of lipid biosynthesis (Nunnari and Suomalainen, 2012).

In this review, we summarize how live cell analysis using fluorescent reporter molecules and (semi)quantitative microscopy can be used to analyze mitochondrial phenotypes. We also discuss various cellular models of mitochondrial disease with respect to their benefits, disadvantages, technical applicability and appropriateness as a disease model. Finally, we present a collection of high-content microscopy strategies to evaluate mitochondrial contribution to disease and to perform drug toxicity and efficacy screening.

## Live Imaging of Cell-Based Readouts to Measure Mitochondrial Functions

Various experimental methodologies quantify mitochondrial dysfunction by focusing on activity measurements of specific mitochondrial enzymes and/or pathways following tissue/cells homogenization and/or using isolated mitochondria (Picard et al., 2011). By contrast, live-cell microscopy assays have the advantage to visualize and quantify functional and structural (sub)cellular (spatial dimension) components *in situ* in living cells. Moreover, microscopy uniquely allows for simultaneous time-lapse monitoring (temporal dimension) and (semi)quantitative measurements of multiple parameters by multispectral imaging (spectral dimension). In particular, developments in fluorescent reporter technology tremendously boosted the use of light microscopy for cell biology studies (Sbalzarini, 2016).

A limitation of fluorescent microscopy is the potential induction of phototoxic stress, which can be caused by illumination of the reporter molecules. Moreover, fluorophores themselves can perturb the physiological function of biomolecules and are subjected to photobleaching. Furthermore, due to calibration limitations, quantification of cellular parameters using single wavelength dyes can be challenging and, in some cases, only relative and qualitative measurements are possible. The application of ratiometric dyes, when possible, takes care of variable dye loading and extrusion responding with a (semi)quantitative change in fluorescence upon target binding. A drawback of the ratiometric dyes is related to their portability to high-throughput where doubling data dimension can create acquisition, storage and processing issues. Implementing ratiometric dyes in multispectral assays can be also inconvenient because of the wavelength limitation.

When mitochondrial contribution to disease is evaluated in living cells, we consider mitochondrial morphology and membrane potential, ROS, ATP and mitochondrial respiration crucial indicators of mitochondrial health status. Their compatibility with fluorescence microscopy assays will be presented in the next paragraphs and is summarized in Table 1.

**Table 1:** Live imaging cell-based mitochondrial readouts and probes.

Readouts and probes	Pros and cons	A	B	C	D	E
**Mitochondrial morphology and Δψ**						
TMRM (or TMRE)	Pros: fast equilibration, low non-specific bindings, low ETC inhibition, low toxicity.	c	m	553	576	Iannetti et al., 2016
rhod 123	Pros: can be used in quenching mode for fast resolving studies to monitor acute changes in Δψ.	c	m	507	529	Perry et al., 2011
DiOC6(3)	Cons: non-specific binding.	c	m	489	506	Perry et al., 2011; Zorova et al., 2018
JC-1	Pros: JC-1 aggregates emit at different λ discriminating high and low Δψ. Cons: Inconsistent experimental data.	c	d	498	525/595	Zorova et al., 2018
MitoTracker	Pros: retained after cell fixation. Cons: not suitable for Δψ live monitoring.	c	m	^∗^	^∗^	Chazotte, 2011
**ROS**						
CM-H2DCFDA	Cons: target aspecificity, no subcellular targeting.	c	m	495	529	Chen et al., 2010
DHE	Cons: target aspecificity, no subcellular targeting.	c	m	480	520	Zielonka and Kalyanaraman, 2010
MitoSOX	Pros: mitochondrial localization. Cons: target aspecificity.	c	m	510	580	Zielonka and Kalyanaraman, 2010
BODIPY 581/591 C11	Pros: intracellular membrane lipid targeting.	c	d	500/650	510/665	Pap et al., 1999
MitoPerOx	Pros: BODIPY 581/591 C11 properties with mitochondrial localization and faster equilibration.	c	d	580/600	590/520	Prime et al., 2012
rxYFP	Cons: pH sensitivity, target aspecificity.	g	m	513	527	Meyer and Dick, 2010; Pouvreau, 2014
roGFP	Pros: minor pH sensitivity than rxYFP, possibility to perform kinetic studies for long-lasting redox changes.	g	d	400/480	510	Meyer and Dick, 2010; Pouvreau, 2014
HyPer	Pros: specific indicator of H_2_O_2_. Cons: pH sensitivity.	g	d	420/500	516	Meyer and Dick, 2010; Bilan and Belousov, 2016
**ATP**						
ATeam	Cons: phototoxicity during long-time observation, pH sensitivity.	g	d	435	527/475	Imamura et al., 2009
BTeam	Pros: increased detection sensitivity, reduced phototoxicity for long kinetic measurement than ATeam.	g	d	460	455/527	Yoshida et al., 2016
ARP-1	Pros: pH independent, higher sensitivity, higher selectivity to distinguish ATP from its analogs.	c	m	500	557	Sunnapu et al., 2017
RSL+	Pros: mitochondrial localized, higher sensitivity, higher selectivity to distinguish ATP from its analogs.	c	m	520	583	De la Fuente-Herreruela et al., 2017


*General pros of genetic probes are: specifically targeted to different subcellular locations. General con of genetic probes is: transfection is required. General pros of ratiometric dual-wavelengths probes: quantitative measure is possible. A. Type of probe: Chemical (c) or genetic (g), B. mono-wavelength (m) or dual-wavelength (d). C. Maximal excitation wavelength. D. Maximal emission wavelength. E. References. ^∗^The MitoTracker^®^ family includes several dyes with different spectral properties (e.g., MitoTracker Red Ex: 579, Em: 599; MitoTracker Green Ex: 490 Em: 516). λ = wavelength.*

### Mitochondrial Morphology and Δψ

Mitochondrial dysfunction is often associated with simultaneous aberrations in mitochondrial morphology (e.g., fragmentation, roundness) and membrane potential (Δψ). Fluorescence live-cell imaging is the most direct method for assessing their spatiotemporal dynamics (Koopman et al., 2008). Different lipophilic cell-permeant, cationic and fluorescent molecules have been presented, which diffuse across the plasma membrane of the cell and accumulate in the mitochondrial matrix in a Δψ dependent manner. These molecules include tetramethylrhodamine methyl ester (TMRM), tetramethylrhodamine ethyl (TMRE) ester, rhodamine 123, DiOC6(3) (3,3′- dihexyloxacarbocyanine iodide), JC-1 (5,5′,6,6′-tetrachloro-1,1′,3,3′-tetraethylbenzimidazolylcarbocyanine iodide), and the MitoTracker^®^ family. Among these molecules, TMRM was described to be the least toxic, the fastest in equilibrating across membranes, and displaying the lowest non-specific localization (Nicholls, 2012; Zorova et al., 2018). Therefore in our research we generally use TMRM to simultaneous analyze mitochondrial morphology and Δψ referred to as mitochondrial morphofunction (Koopman et al., 2008; Iannetti et al., 2016). The cell types, staining, imaging conditions and descriptors applicable for the analysis of mitochondrial morphofunction have been previously reviewed (Iannetti et al., 2015; Zorova et al., 2018) and are summarized in our recent study (Iannetti et al., 2016). To technically validate measurements of Δψ using TMRM, we performed oligomycin/bongkrekic acid, rotenone and FCCP acute injections while kinetically measuring mitochondrial TMRM fluorescence fluctuations (Iannetti et al., 2016). Although TMRM measurement, even under highly standardized experimental settings, have been considered still semi-quantitative (Leonard et al., 2015; Nicholls, 2018) attempts using this dye to perform more absolute measurements have been performed combining it with the analysis of the plasma membrane potential (Gerencser et al., 2016).

Protein-based probes targeted to the mitochondria, such as mito-GFP, are also a valid tool to study mitochondrial morphology and dynamics (Rizzuto et al., 1995; Nomura et al., 2009), however, these do not allow the simultaneous study of Δψ.

### Reactive Oxygen Species

Reactive oxygen species (ROS) is a general term that includes both oxygen radicals and non-radical agents that can be easily converted into radicals (Halliwell and Gutteridge, 1985). ROS are generated both in the cytosol and in mitochondria as (by) products of normal physiological cell metabolism (Murphy, 2009; Forkink et al., 2010). Depending on the chemical nature of the ROS, the location at which they are generated and their (local) concentration, ROS can exert a signaling role or induce oxidative and/or redox stress (Lin and Beal, 2006; Smeitink et al., 2006) emphasizing the importance to determine their concentration, types, and localization with precision (Woolley et al., 2013).

Several non-microscopy based approaches are available (e.g., mass spectrometry, western blotting, and immunohistochemistry) to indirectly study ROS via the quantification of the accumulated reaction products (oxidized protein, lipid, and DNA) (McDonagh, 2017; Teixeira et al., 2018). Due to this accumulation these methods have an high sensitivity, however, they do not consider the spatial and temporal dimensions because cell lysates are usually analyzed at end points.

A wide range of chemical or proteinaceous fluorescent ROS probes has been developed (Zhang and Gao, 2015). The two most commonly used chemical ROS probes are 5-(and-6)-chloromethyl-2,7-dichlorodihydrofluorescein diacetate (CM-H_2_DCFDA) (Chen et al., 2010) and dihydroethidium (DHE) (Zielonka and Kalyanaraman, 2010). While CM-H_2_DCFDA was initially developed and used to specifically detect H_2_O_2_ and DHE for superoxide detection, growing evidence indicates that these are both non-specific ROS indicators that should be used for qualitative analysis of total cellular oxidant stress rather than for specific ROS types (Koopman et al., 2006; Chen et al., 2010; Zielonka and Kalyanaraman, 2010). Despite that, they currently are the most popular ROS sensors because of their technical ease of use that no alternatives can yet guarantee. Especially for CM-H_2_DCFDA a rigorous monitoring of the experimental setup and in particular of the protection from environmental light, which is more easily executed with automated imaging, is required. Under tight quality controlled conditions these dyes can produce meaningful and robust qualitative information related to intracellular ROS bursts.

MitoSOX, which is DHE linked to a TPP moiety for rapid accumulation in mitochondria, would ideally allow detection of mitochondria specific ROS production (Robinson et al., 2006). However, this dye needs to be used with caution, since oxidation of the probe may have happen before it enters the mitochondria (Connolly et al., 2017). Furthermore, the oxidized probe tends to bind to DNA upon which its fluorescence is much increased (Mukhopadhyay, 2008). In our experiments we take especial care to monitor the acquired images and exclude all data in which the staining is not strictly mitochondrial (Beyrath et al., 2018). Flow cytometric and plate reader experiments using mitoSOX should therefore be avoided or interpreted with great care.

Lipid peroxidation of the mitochondrial inner membrane represents a major cause of mitochondria disruption (Morris et al., 2018; Nielson and Rutter, 2018) and ferroptosis cell death (Yang and Stockwell, 2016) and it is also considered a crucial readout for evaluation of mitochondrial dysfunction. A ratiometric fluorescent probe, MitoPerOx, specific for mitochondrial fatty acid peroxidation was developed (Prime et al., 2012). MitoPerOx is the mitochondrial targeted version of the BODIPY 581/591 C11 used for the measurement of peroxyl radicals in the general cellular membrane fraction (Pap et al., 1999).

The major drawbacks of the currently available chemical fluorescent ROS probes are the non-specific photo- and chemical-oxidation and the limited availability of subcellular targeting options. This often causes an unclear temporal resolution dynamic: it is not clear where the oxidation of the probe happened. To overcome this limitation, genetically encoded ROS indicators have also been developed: redox-sensitive yellow fluorescent proteins (rxYFP family), redox-sensitive green fluorescent proteins (roGFP family) and the H_2_O_2_ probe HyPer (Pouvreau, 2014). The working principle of rxYFP and roGFP is based on a change in the oxidation state of the redox-reactive cysteines group that induces a conformational change in the fluorescent properties of the sensor protein (Meyer and Dick, 2010). HyPer instead works by an H_2_O_2_-sensing regulatory domain of a prokaryotic transcription factor which cysteine active site readily reacts with H_2_O_2_ inducing a conformational change of the fluorescent protein (Bilan and Belousov, 2016). These genetically encoded ROS and redox indicators have the advantage of providing more reliable real-time monitoring of specific ROS in subcellular compartments. However, using genetically encoded chimeric proteins that require cell transfection and gene expression, can be technically challenging depending from the cell type (Kim and Eberwine, 2010).

### ATP

ATP plays a central role in bioenergetics and intra/inter-cellular signaling. It can be considered an indicator of cellular and mitochondrial health status (Koopman et al., 2012). A number of well-established assays such as HPLC-based methods and biochemical assays based on luciferase-luciferin bioluminescence guarantee high specificity and accuracy to measure ATP (Vives-Bauza et al., 2007), however, these approaches are not applicable to study living cells because they require the physical extraction of the ATP from the cells by cell homogenization procedures.

Currently, luciferin-luciferase bioluminescence assays using plate readers represent still the gold standard to measure ATP in cells and robust kits are commercially available from different providers.

Significant advances have been made to image ATP using fluorescent, chemiluminescent, bioluminescent and resonance energy transfer technologies based on genetically encoded or chemical probes. Genetic approaches provide great flexibility in the subcellular localization to be targeted. In particular the fluorescence resonance energy transfer (FRET) sensors of the ATeam family (Imamura et al., 2009) that consist of a subunit of the bacterial F_o_F_1_-ATP synthase combined with fluorescent proteins of different colors, were developed to differentially target cytosol, nucleus or mitochondrial matrix and have been validated in several studies (Liemburg-Apers et al., 2011; Forkink et al., 2014). BTeam the next generation genetically encoded sensors to image ATP, have increased detection sensitivity and allow kinetic measurement of cytosolic ATP levels of the same cells (Yoshida et al., 2016).

Chemical probes that passively diffuse into the cells, such as the rhodamine-based chemical sensors, ARP-1 and RSL^+^, have been recently developed for real-time imaging of mitochondrial ATP in living cells (De la Fuente-Herreruela et al., 2017; Sunnapu et al., 2017).

To the best of our knowledge, no chemical ATP probes are currently commercially available. Because ATP concentrations vary widely among tissues, cells and subcellular compartments and because local concentrations vary on a millisecond timescale, the availability of probes with a variety of ATP affinity ranges, fast ATP binding and response kinetics seem features necessary for future ATP probes (Rajendran et al., 2016).

### Mitochondrial Respiration

In the ETC the production of ATP is directly coupled to the consumption of molecular oxygen (O_2_). Quantifying intracellular O_2_ consumption (respirometry) is therefore a direct estimate of the mitochondrial respiratory activity and as such another crucial readout for the mitochondrial and cellular health status (Brand and Nicholls, 2011). The classical respirometry approaches are electrode-based systems like the Seahorse^®^ XF Analyzers (Smolina et al., 2017) and the Oroboros O2k-Fluo Respirometer (Makrecka-Kuka et al., 2015). These are highly sensitive devices to quantify intracellular oxygen levels in living cells. However, these approaches lack spatial resolution to distinguish O_2_ concentration at the single cell or subcellular level.

Imaging approaches using microscopy have been developed also for the quantification of O_2._ These are mostly based on oxygen induced quenching of phosphorescence or luminescence generated by cell-permeable probes that all still are in an experimental phase. Extensive description of the pitfalls, advantages and opportunities were summarized by Dmitriev and Papkovsky (2015) and more recently by Yoshihara et al. (2017).

Despite all the research, further development is still necessary before O_2_ imaging will be available for routine use (Dmitriev and Papkovsky, 2015). Therefore, we still consider electrode-based systems the benchmark to quantify intracellular O_2_ consumption.

## Human Cell Models in Mitochondrial Research

The choice of an appropriate cell model, recapitulating robust pathological read-out appears to be the first challenge and goal to achieve (Breuer et al., 2013) when evaluating mitochondrial contribution to disease.

Because cell specific metabolism is highly regulated at the genetic, transcriptional and post transcriptional level, every cell type has different energetic requirements. Therefore, mitochondrial physiopathology differs substantially between different cell models depending from their tissue of origin (Smeitink et al., 2006). Mitochondrial functionality can be differentially regulated, induced or even suppressed also depending from environmental factors such as nutrient availability, oxygen condition, differentiation, passage number and many other variables that should therefore be tightly controlled (Benard et al., 2010).

The human cell models most commonly used in mitochondrial research, with their advantages and disadvantages are discussed in the next paragraphs and listed in Table 2.

**Table 2:** Human cell models used in mitochondrial research.

Pros		Cons
**Primary fibroblasts**	- relatively easy availability from patients and matched controls - easy isolation - robustness in culture, storage, and transport - low cost - not genetically modified - flat morphology and a relatively large size allow non-confocal imaging - present the biological aging of the patients and are usually sampled at the moment of diagnosis	- do not originate from the defective organs - stress conditions are often necessary to enhance pathological symptoms - slow proliferation - require large surface to grow significant number of cells - can be used only for a limited number of passages
**Cybrids**	- relationship between mtDNA and phenotype can be studied - robustness in culture, storage, and transport - low cost	- nuclear-mitochondrial interactions are lost - cancer-like glycolytic bioenergetics profile - genetics aberrations - useful only to study mitochondrial encoded mutations
**iPSc and iPSc-derived**	- mimic the defective organs - originate from the specific patient - pathological symptoms are usually present - derived from easily accessible patient material (skin, blood, urine) - can be differentiated into virtually any cell type of the body - open possibilities for precision medicine approaches	- genetically modified: mutagenesis risk - suboptimal standardization - mtDNA mutations can impair cellular reprogramming to iPSCs and differentiation - costly and timely procedure - iPSc heterogeneity can mask actual disease-associated phenotypes - low yield of differentiated cells


### Primary Culture

Primary cells are a good model to study disease-related phenotypes, since they do not undergo genetic manipulation, present the biological aging of the patients and are usually sampled at the moment of diagnosis. When using patients-derived primary cells, the use of adequate control cells to compare and normalize data to is crucial. The common approach is to use samples derived from healthy gender- and age-matched volunteers. However, because of the different genetic backgrounds between any two individuals, cell-specific non-pathological differences may be introduced. This makes comparison of multiple control and patient cell lines in parallel essential.

In mitochondrial research, the most used primary cell model is skin fibroblasts. A review by Auburger et al. (2012) highlighted some of the advantages and drawbacks of primary skin fibroblast cultures. Easy availability from patients and matched controls and robustness in culture, storage, and transport were identified as some of the main advantages. In relation to microscopy, fibroblasts are also ideal for imaging simply by epifluorescence (non-confocal) microscopy. Thanks to their extremely flat morphology and a relatively large size, imaging one single focal plane is sufficient to have most of the cell body in focus. However, in mitochondrial disease, the cells with high bioenergetic requirements and concomitant reliance on mitochondrial ATP generation such as neurons, retinal, muscle cells, and in particular cardiomyocyte are often the most affected and often associated with malfunction of the corresponding organs (Breuer et al., 2013; Liang et al., 2014). Therefore, one of the possible concerns using fibroblasts in mitochondrial research regards the fact that these cells do not originate from a defective organ. Indeed, despite the presence of the pathogenic mutation, aberrant phenotypes are not always observed in primary fibroblasts in classical culture regimes. Therefore, different stress conditions have been used by the research community to enhance pathological symptoms and study the dysfunctions. Culture medium in which galactose replaces glucose to enforce ATP production to rely on mitochondrial metabolism (Robinson et al., 1992; Rossignol et al., 2004; Iannetti et al., 2018) and buthionine sulfoximine treatment to reduce glutathione, the main cellular antioxidant (Shrader et al., 2011; Beyrath et al., 2018), are two well-known strategies to induce or enhance pathological mitochondrial phenotypes.

### Cytoplasmic Hybrids (Cybrids)

Cytoplasmic hybrids (cybrids) are generated by fusing nuclear-depleted cells derived from patients carrying mtDNA mutations with cell lines in which the mtDNA has been removed (so called Rho Zero Cells). The cybrids model has been traditionally used to study mtDNA mutations and dissect the relationship between mtDNA and phenotype alterations (King and Attardi, 1989; Swerdlow, 2007; Wilkins et al., 2014). By introducing the patient-derived mtDNA into a healthy nuclear background, it is possible to dissect whether a certain mutation is sufficient to cause bioenergetics or cellular defects, making cybrids instrumental in dissecting the precise cellular and molecular consequences of a specific mtDNA mutations and the field of mtDNA-related diseases has greatly benefitted from this model.

Unfortunately, the patient-specific interplay between mitochondrial and nuclear genomes, which may play a contributing role in the OXPHOS dysfunction manifestation (Spinazzola and Zeviani, 2009), is lost in the cybrids model. Another disadvantage of cybrids, like other common immortal cell lines, is that they usually present genetic aberrations and relay on a glycolytic bioenergetics and not on OXPHOS like disease affected cells (Abramov et al., 2010). Moreover, cybrids are useful only to study mitochondrial encoded mutations and not for nuclear ones.

### iPSCs and Differentiated iPSCs-Derived Cells

Human induced pluripotent stem cells (iPSCs) are obtained from somatic cells through the process of cellular reprogramming (Takahashi et al., 2007). iPSCs can be derived from easily accessible patient material (skin, blood, urine) and can be coaxed to differentiate into virtually any cell type of the body. Diseases affecting the nervous system, like mitochondrial disorders, may particularly benefit from iPSC research, since the affected patient tissue is not readily available for testing. Genome editing techniques, such as CRISPR/Cas9, combined with iPSCs technology have opened unprecedented opportunities in manipulating nDNA to induce or correct specific mutations of interest. It is possible to generate isogenic iPSCs, which carry the same background (from a control or a patient individual) and differ only in one single disease-causing mutant gene (Grobarczyk et al., 2015). However, genome editing technology is well established only for nuclear DNA (Komor et al., 2017), as engineering of mitochondrial DNA still remain technically challenging (Patananan et al., 2016; Gammage et al., 2017). iPSCs can be applied in compound screens aimed at identifying treatments for mitochondrial diseases (Inak et al., 2017). In fact, one of the key advantages of iPSC-based models is that they may allow a precision medicine approach (Gibbs et al., 2018).

On the other hand, iPSCs also hold disadvantages. Some studies reported that mtDNA MELAS mutations impair cellular reprogramming to iPSCs (Yokota et al., 2015). Cellular fate-determination processes may also be affected, in particular neuronal and cardiac lineage commitment (Folmes et al., 2013; Hatakeyama et al., 2015; Yokota et al., 2017). This may be regarded as a possible readout for mitochondrial dysfunction, but also as a technical complication to generate patient iPSC derived cell lines. Furthermore, the generation of iPSCs is costly and time consuming. It is now apparent that different iPSC lines can be very heterogeneous, thereby masking actual disease-associated phenotypes. Unfortunately, the reprogramming process itself can also induce nuclear and mitochondrial DNA alterations (Pera, 2011; Perales-Clemente et al., 2016), and therefore the genome of all iPSC lines needs to be carefully monitored. The differentiation of iPSCs is time-consuming and often very challenging in obtaining robust and homogenous differentiated progeny (Saha and Jaenisch, 2010), resulting in a small number of obtained differentiated cells that can limit the scalability and the high-throughput applications of iPSC-derived cells. Finally, given that iPSCs rejuvenate the state of mitochondria (Lisowski et al., 2018) and the aging-associated epigenetic signature (Mertens et al., 2018), it has been suggested to circumvent the generation of iPSCs by using a direct reprogramming approach (Vierbuchen et al., 2010). In this approach, patient-derived fibroblasts can be directly converted into neurons without going through the state of iPSCs, thereby retaining the aging signature (Mertens et al., 2015; Victor et al., 2018). Nonetheless, also directly reprogrammed cells carry disadvantages as they need to be generated newly continually and cannot be easily used for genome editing.

## High-Content Screening Applications to Study Mitochondrial Functions

High-content screening (HCS) is defined as a cell-based phenotypic approach where readouts are imaged by multiplexed and automated microscopy (Zanella et al., 2010; Pegoraro and Misteli, 2017); this is also referred to as cellomics (Taylor, 2007).

Because of the fast developments of technologies, probes and applications and the upcoming field of iPSCs technology generating faithful cell disease models, the field of cellomics is now on the brink of catching up with the other–omics approaches.

Already in 2007 an HCS method was developed combining Δψ analysis with other cellular parameters measured in human liver carcinoma cells (HepG2) grown in a microfluidics device (Ye et al., 2007). Also performed in HepG2 cells an HCS assays has been described to screen drugs based on six parameters among which Δψ and mitochondrial area (Persson et al., 2013) or intracellular redox state (Ye et al., 2007; Donato et al., 2012). A cellomics liver toxicity assay using iPSC-derived hepatocytes was recently published that focuses on drug development and toxicity testing, studying mitochondrial parameters as indicators of cellular health (Sirenko and Cromwell, 2018). Leonard et al. addressed more technical aspects of HCS application development combining the quantitative analysis of mitochondrial morphology and Δψ in living photoreceptor cells with supervised machine learning (Leonard et al., 2015). In 2016 we described a detailed protocol to optimize multiplexed high-content analysis of mitochondrial morphofunction in primary human skin fibroblasts showing potential implementation of the protocol to HCS format (Iannetti et al., 2016). With this same cell type a high-content method was also developed to combine the study of cellular ROS levels and mitochondrial morphofunction (Sieprath et al., 2016). Other cellomics applications focused instead primarily on the identification of mitochondrial pathological phenotypes to evaluate their contribution to disease. Among these, a strategy based on iPSC-derived NPCs of mtDNA patients in which a Δψ–related pathological phenotype was identified and used to screen a compound-library (Lorenz et al., 2017). A 384-well plates based HCS application, identifying reduced Δψ and mitochondrial morphology aberrations in iPSC-derived neurons from Parkinson’s disease patients as compared to controls, was also recently published (Little et al., 2018).

All these studies show the applicability of mitochondrial morphofunction and ROS analysis as robust HCS/cellomics applications. These will likely in the near future be complemented with fluorescent imaging technologies to detect ATP and oxygen consumption, which, to the best of our knowledge, have not been explored yet.

## Conclusion

Cellomics represents a powerful technology, already established in many research labs, to study in an unbiased manner mitochondrial functions monitoring multiple readouts, portable to different cells models and able to provide valuable insight in mitochondrial phenotypes and their contribution to disease.

## Author Contributions

All authors listed have made a substantial, direct and intellectual contribution to the work, and approved it for publication.

## Conflict of Interest Statement

EI, JB, and HR are full-time employees of the SME Khondrion (www.khondrion.com). JS is the founding CEO of Khondrion. WK is a scientific advisor of Khondrion, Mitoconix Bio Ltd. (Ness Ziona, Israel) and Fortify Therapeutics, Inc. (Palo Alto, CA, United States). The remaining author declares that the research was conducted in the absence of any commercial or financial relationships that could be construed as a potential conflict of interest.
